# Dorsal Augmentation Rhinoplasty by Cartilage Allograft

**DOI:** 10.1111/jocd.16724

**Published:** 2024-12-12

**Authors:** Armen Harutyunyan, Gagik Hakobyan

**Affiliations:** ^1^ Plastic Surgeon, Astkhik Medical Center, Department of Oral and Maxillofacial Surgery Yerevan State Medical University After M. Heratsi Yerevan Armenia; ^2^ Yerevan State Medical University After M.Hertsi Yerevan Armenia

**Keywords:** allogeneic cartilage, dorsal nasal rhinoplasty, nasal septum

## Abstract

**Background:**

The aim of this study was to evaluate long‐term outcomes and patient satisfaction after nasal dorsal rhinoplasty with allogeneic cartilage graft of the nasal septum.

**Materials and Methods:**

The present study included 104 patients (41 males and 63 females) whose mean age at surgery was 28.7 years and who underwent nasal dorsal augmentation with allogenous nasal septal cartilage graft during the period 2017–2023.

Depending on the use of platelet‐rich fibrin (PRF), the patients were divided into two groups:

- Group I had PRF applied in dorsal rhinoplasty and included 53 patients (29 males and 24 females).
- Group II (control group) had dorsal rhinoplasty without the use of PRF and included 51 patients (26 males and 25 females).

Donor patients included those who had taken excess nasal septal cartilage during functional rhinoplasty. According to the indications, crushed allogenic septal cartilage graft and PRF were also used. Esthetic results of rhinoplasty were assessed from preoperative and postoperative clinical pictures and also by visual examination. To assess the results of esthetic rhinoplasty, the Portuguese version of the Utrecht Questionnaire (UQ) was used, which contains a visual analog scale (VAS) on a 5‐point Likert scale.

**Results:**

In patients included in the study, there were no serious complications: there was no deformation or extrusion, and allograft resorption was not observed. After rhinoplasty in the Group I patients, the mean score of VAS esthetic satisfaction with nose improved from 3.6 preoperatively to 8.5 3 months postoperatively and 9.2 12 months postoperatively. After rhinoplasty in the Group II patients, the mean score of VAS esthetic satisfaction with nose improved from 3.7 preoperatively to 7.8 3 months postoperatively and 8.3 12 months postoperatively.

**Conclusions:**

Rhinoplasty with allogeneic cartilage graft of the nasal septum allows to achieve stable, positive, functional, and esthetic results and is safe to use. It is an alternative to autologous cartilage used in rhinoplasty and also prevents additional surgical procedures.

**Abbreviations**
CT: computed tomography
PRF: platelet‐rich fibrin
UQ: Utrecht questionnaire
VAS: visual analog scale

## 1 Introduction

The proportions of the nose are critical for good facial esthetics and nasal esthetic disorders are most often caused by congenital malformations, trauma, dermatological diseases, and so forth. One of the most popular plastic surgeries in the maxillofacial area is rhinoplasty [1]. The main goal of rhinoplasty is to provide the patient with a clean nasal passage for the airway, a cosmetic effect to increase self‐confidence and improve the quality of life. Among the defects of the nose, the saddle type of the nose is often found, which is associated with the loss of the dorsal part of the quadrangular cartilage [2]. In patients with a saddle nose, the esthetics of the face are disturbed, which makes the patient in turn to go for the rhinoplasty method. Over time, this leads to a saddle deformity, retraction of the columella, and a decrease in the projection of the tip of the nose.

In dorsal rhinoplasty, surgical and non‐surgical methods are used [3, 4]. These methods include different materials such as autologous bone grafts (septum, choncal, 7–11 ribs, etc.), fluid cartilage, and regenerative strategies such as fat grafting, PRP, and rhinofiller [5].

Autologous materials are considered as the best material and remain the gold standard because they are more resistant to infection, integrate well with the surrounding tissues, and undergo minimal resorption [6, 7].

Histologically, cartilage consists of the ground substance and fibers of collagen and elastin; chondrocytes are located in the lacunae of the extracellular matrix [8, 9]. On the basis of the available scientific literature, believed that the immune system plays a limited role in the recognition and rejection of cartilage allografts, avascularization determines the “immune privilege” of cartilage [10].

The main role of the immunological activity of cartilage is played by chondrocytes, which express antigens and provoke an important immune response, whereas the cartilage matrix does not express major histocompatibility complex antigens [11].

The choice of graft material depends on the size of the defect. For mild saddle nose, a cartilaginous septum or concha cartilage is used because it is strong and flexible, but in many patients, the amount of septal or concha cartilage is insufficient to achieve reconstruction in patients with saddle noses [12].

In augmentative rhinoplasty, surgeons often require enough graft for dorsal augmentation, the preferred graft material is autogenous costal cartilage, iliac crest graft which is available in large quantities, sufficient strength to provide adequate support [6, 13, 14, 15].

However, due to the high risk of donor diseases, the risk of complications such as pneumothorax, postoperative pain, and scarring, many specialists are limited of its use [16].

In minor contour deformities of the nasal surface, liquid cartilage is used. Liquid cartilage grafts are more flexible for filling lesions, and it is used in microinvasive procedures and minor deformities of nose. Injectable cartilage is prepared from septal, lateral, or less commonly conchal cartilage and is injected through a 14‐ to 18‐gauge needle or a 1.3‐ to 1.7‐mm lipofilling blunt‐tipped cannula from the skin or mucosal surface into the defect area without any incision or elevation of skin flap. The long‐term clinical outcome of the study by Manafi et al. [17] supports the conclusion that the cartilage microstructure is not degraded when prepared as an injectable cartilage shave.

Its disadvantage is poor mechanical stability and rapid decomposition, leading to its wrinkling and deformation [18].

Currently, autologous fat is often used during dorsal rhinoplasty to improve the esthetics of the nose. Nasal lipofilling is one of the minimally invasive corrections for nose esthetics and contour correction. This method is effective with a low complication rate [19]. Platelet‐rich fibrin (PRF) is also currently widely used in rhinoplasty. There exist studies on the use of PRF alone in dorsal rhinoplasty without cartilage or mixed with high‐density fat [20, 21].

Although autograft cartilage remains the gold standard in nasal dorsum rhinoplasty [22, 23], in some clinical cases, cartilage allograft is preferred.

The focus has been shifted toward the use of allogenic cartilage scaffolds in recent years due to their large availability of resolving the continuous growing augmentative rhinoplasty [24]. Numerous surgeons have reported their results of using allogeneic cartilage, treated with different processing methods in rhinoplasty. The use of allogeneic cartilage grafts in augmentation rhinoplasty has been continuously increasing [25], which is due to the fact that these grafts are devoid of many of the above‐mentioned disadvantages and the additional operation time associated with the collection of autocartilage.

Allografts have high osteoconductivity and are the prototype of the osteoconductive matrix, and the proliferation of granulation tissue will lead to revascularization of the graft [26].

In this context, allogeneic cartilage grafts for rhinoplasty may be an attractive surgical option [24]. Allogeneic cartilage is treated with high doses of ionizing radiation, osmotic degradation, freeze‐drying, and chemical sterilization, either alone or in combination [25].

Allogeneic material is sterilized most often by gamma rays from 30 000 to 60 000 Gy. With an increase in the dose, the rigidity of the cartilage decreases and the intensity of subsequent resorption increases [27].

The high difference in assessing the effectiveness of the use of the allogeneic graft is due to two main factors: processing and conservation of allogeneic material.

Allogeneic grafts have shown good results in dorsal rhinoplasty because they are strong enough to provide structural support and overcome soft tissue resistance [28, 29].

Allogeneic human dermis from AlloDerm (LifeCell Corporation, Branchburg, NJ) is also a good alternative to autogenous material for dorsal rhinoplasty as a biocompatible, nonimmunogenic, readily available, and relatively affordable material [30].

However, allogeneic cartilage also has their limitations. Allogeneic cartilage has amazing antigenic properties, which is determined by the expression of antigens from the structural components of the cartilage tissue, and the perichondrium has a relatively lower immunogenicity [31].

As possible complications allogeneic cartilage, the risk of inflammatory reactions, infection or partial lysis of the graft with its subsequent displacement has been [32].

In numerous augmentation procedures, rhinoplasty specialists also use alloplastic materials (including polytetrafluoroethylene, silicones, and polyethylenes) to reconstruct the nasal dorsum because they are affordable, prevent donor site complications, and are preferred by patients [33, 34]. However, the use of alloplasty materials for rhinoplasty is not without its disadvantages; in particular, various postoperative complications may occur (infection, protrusion, and displacement) [35, 36].

Currently, liquid rhinoplasty using dermal fillers has gained popularity. The use of new hyaluronic acid (HA) dermal fillers shows great promise in non‐surgical rhinoplasty (NSR) and is becoming more popular due to their good efficacy and safety, lower risk of complications, and shorter treatment time [37, 38, 39, 40]. However, it seems that it is a temporary result, requiring only a short period of time with minimal recovery time.

According to the literature, up to 96.5% of the population has in one way or another degree of curvature of the septum of the nose [41]. Currently, corrective operations on the nasal septum are widely common, because it is the partition that is involved in providing normal respiratory function, as well as the formation of esthetic appearance of the nose.

According to Killian and Rudert, one of the most common operations in rhinoplasty is submucosal resection with the removal of a significant portion of the cartilaginous part of the nasal septum [42, 43].

The choice of the ideal graft for dorsal rhinoplasty remains a matter of debate. To date, according to the literature, there is no system for selecting the optimal material for the reconstruction of the dorsal nasal defect, depending on the clinical situation.

When performing operations for esthetic reasons, the auto‐cartilaginous part of the nasal septum often serves as a source of plastic material necessary for performing the operation [44].

One of the alternative solutions may be the technique we propose. The resected portions of the cartilaginous part of the curvature of the septum of the nose can be successfully used as a nasal septum allogeneic donor graft for patients with a dorsal nasal defect.

Therefore, in this article, we aimed to summarize the benefits and challenges of using an allogeneic nasal septal cartilage graft for dorsal rhinoplasty.

The aim of this study was to evaluate long‐term outcomes and patient satisfaction after nasal dorsal rhinoplasty with allogeneic cartilage graft of the nasal septum.

## 2 Methods

This clinical series study included 104 patients (41 males and 63 females) whose mean age at surgery was 28.7 years and who underwent nasal dorsal augmentation with allogenous nasal septal cartilage graft from 2017 to 2023.

### 2.1 Inclusion Criteria

The inclusion criterion was healthy patients with saddle who were scheduled to undergo a cosmetic surgical procedure involving cartilage transplantation.

### 2.2 Exclusion Criteria

The exclusion criteria were patients with age of 18 years; patients with reconstructive rhinoplasty for tumor or traumatic deformities of the nose; presence of significant endocrine, immunologic, and dermatologic, abnormalities; and history of radiation treatment to the area.

All patients underwent a visual/physical examination and photographs were taken before and after the surgery. The patients were assessed for the profile of the nose and the thickness of the skin. The CT results made it possible to study the structures of the nasal septum and the extent of the existing defect, and to clarify the condition and position of the nasal bones.

All patients were informed about the use of the localized nasal septal cartilage allograft procedure, and then a written and verbal consent was obtained prior to the commencement of procedure.

Donor patients had taken excess nasal septal cartilage during functional rhinoplasty. Donors were tested for hepatitis B, hepatitis C, and HIV prior to the surgery.

Cartilage graft from the septum was subjected to radiation sterilization with gamma beams and conservation in a solution containing thiomersal (white merthiolate) and stored in a refrigerated environment at 4°C.

#### 2.2.1 Cartilage Preparation

**FIGURE 1:**
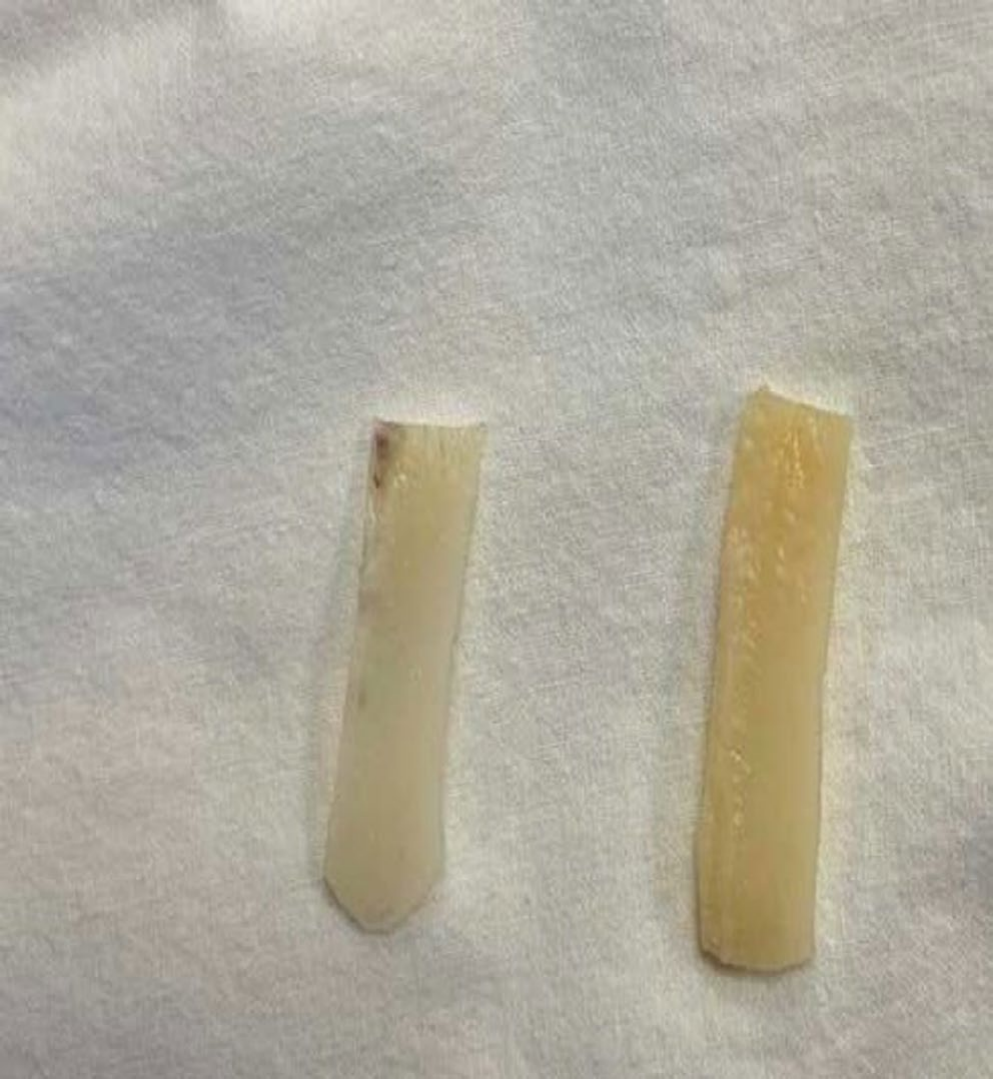
Nasal allocartilage consisted of two parts, vertical and horizontal.

**FIGURE 2:**
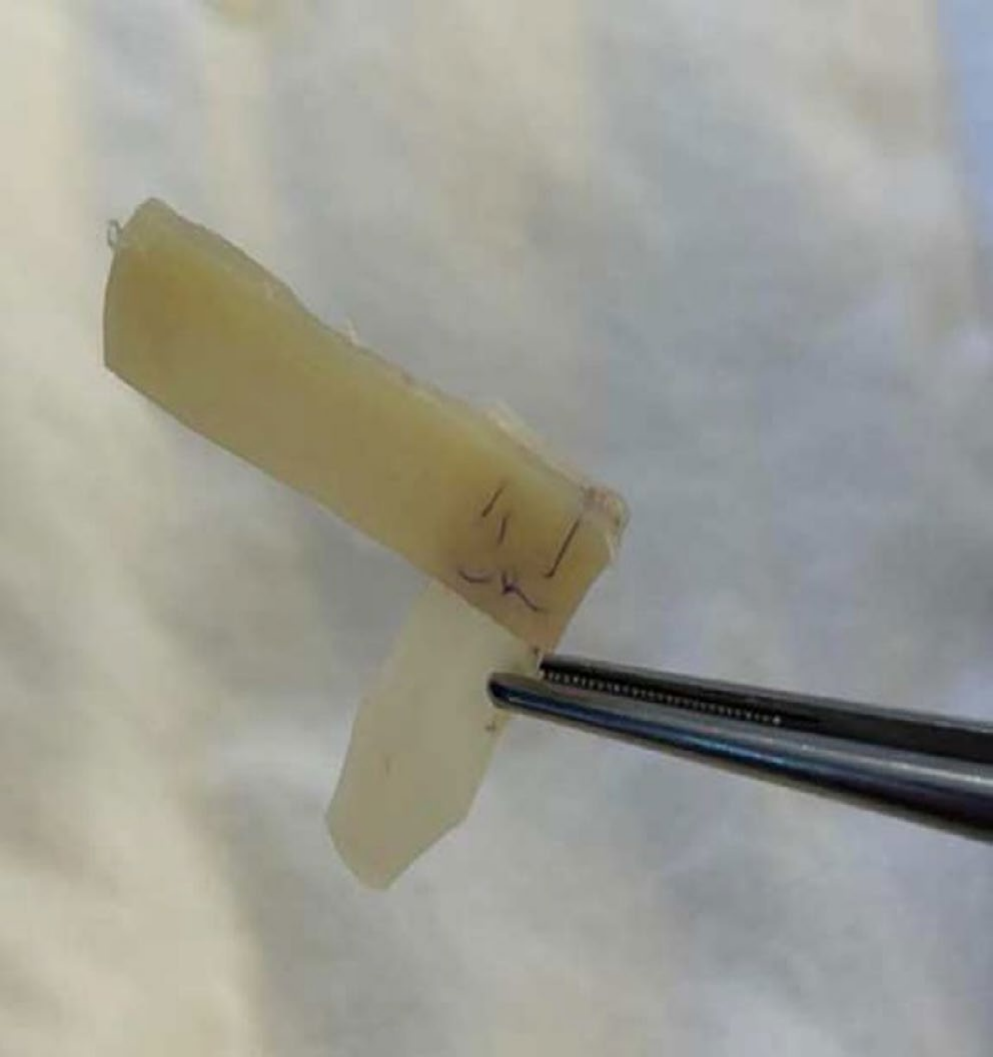
Allocartilage graft in the form of the Latin letter “L” was modeled of two straight fragments of cartilage sewn with absorbable MonoPlus 5/0 thread at right angles.

The allogeneic septal cartilage graft was washed from the preservative with 0.9% sodium chloride solution under sterile conditions before use, so that the perichondrium was completely removed. Cartilage should not have any foci of softening, or foci or calcification. Allocartilage graft in the form of the latin letter “L” was modeled of two straight fragments of cartilage (a vertical part and a horizontal part) with a scalpel and sewn with absorbable Mono Plus 5/0 thread at right angles (Figures 1 and 2). The vertical part acted as a support for the tip of the nose. The size of the vertical part of the graft varied depending on the condition of the anterior superior edge of the patient's quadrangular cartilage, of which it was supposed to be a continuation. The horizontal part was a volume and contour restorer of the nasal dorsum.

According to the indications, with an increase in the nasal dorsum, crushed allogenic septal cartilage and PRF were also used. Depending on the use of PRF, patients were divided into two groups:

- Group I had PRF applied in dorsal rhinoplasty and included 53 patients (29 males and 24 females)
- Group II (control group) had dorsal rhinoplasty without the use of PRF and included 51 patients (26 males and 25 females).

#### 2.2.2 Surgical Procedure

All patients underwent rhinoplasty under general anesthesia with orotracheal intubation and local anesthesia in the form of articaine forte 4% with adrenaline at the site of the intended incision. For esthetic purposes, closed rhinoplasty was performed, degloving incisions were made, and the periosteum and skin were exfoliated along the entire surface of the nasal dorsum.

The septal cartilage allograft was placed in the recipient site at the defect area under the periosteum, part of the allograft on the nasal bones and part on the cartilage of the middle vault. The perpendicular part of the allograft was located in front of the caudal part of the quadrangular cartilage between the medial crura of the inferolateral cartilage. At the end of the operation, the skin flap was returned to its normal anatomical position and closed with 5/0 nylon sutures. The final position of the graft was corrected by external manual manipulations.

Twenty‐three patients with underdeveloped nasal tip underwent nasal tip reconstruction with lift the tip of the nose. To minimize soft tissue edema and graft displacement at the end of rhinoplasty, a dressing (Steri‐Strip) was applied to all of the nasal dorsum for a week; the length of stay in the hospital was 2–4 days. Prophylaxis antibiotic uses a third‐generation cephalosporin; no other treatment is used to suppress the immune system.

The patients' follow‐up was carried out on the day after surgery, every 2 days, and then 2, 6, 12, 24, and 36 months after the surgery.

Esthetic results of dorsal rhinoplasty were assessed from preoperative and postoperative photographs and also by visual inspection. Two images were captured from frontal and lateral views. Resorption, warping, graft displacement, scarring, and infection were also assessed.

For evaluating the results of patients after rhinoplasty surgery, the Portuguese version of the “Utrecht Questionnaire (UQ) for the evaluation of esthetic rhinoplasty results” is a valid tool.

To assess the results of esthetic rhinoplasty, the Portuguese version of the UQ was used, which contains a visual analog scale (VAS) on a 5‐point Likert scale. Patients before and after surgery (after 3rd and 12th month) were assessed for the appearance of the nose and quality of life.

#### 2.2.3 Statistical Analysis

SPSS software (Version 17.0; SPSS Inc., Chicago, Illinois) was used for statistical analyses Comparison of the two groups was performed using Student's *t*‐test, and *p* values < 0.05 were considered statistically significant.

## 3 Results

After dorsal rinoplasty, all patients showed a significant increase in dorsal height, root height, and tip projection compared to preoperative data.

Erythema or allograft‐related infections have not been reported after rhinoplasty. Of the 53 patients of Group I, there was no deformity or extrusion, and no infection or resorption of the allograft was observed. One patient had a leftward deviation of the dorsum at the 6‐month follow‐up and underwent revision (Table 1).

**TABLE 1:** Complications in I and II Groups.

Complications	Group I	Group II
Paresthesia	0	1
Resorption	0	1
Warping	1	2
Depression	0	1
Deviation	1	2
Total	2	7

The patient had satisfying esthetic and functional outcomes at the 12‐month follow‐up with stable structural support and non‐noticeable warping or graft displacement.

**FIGURE 3:**
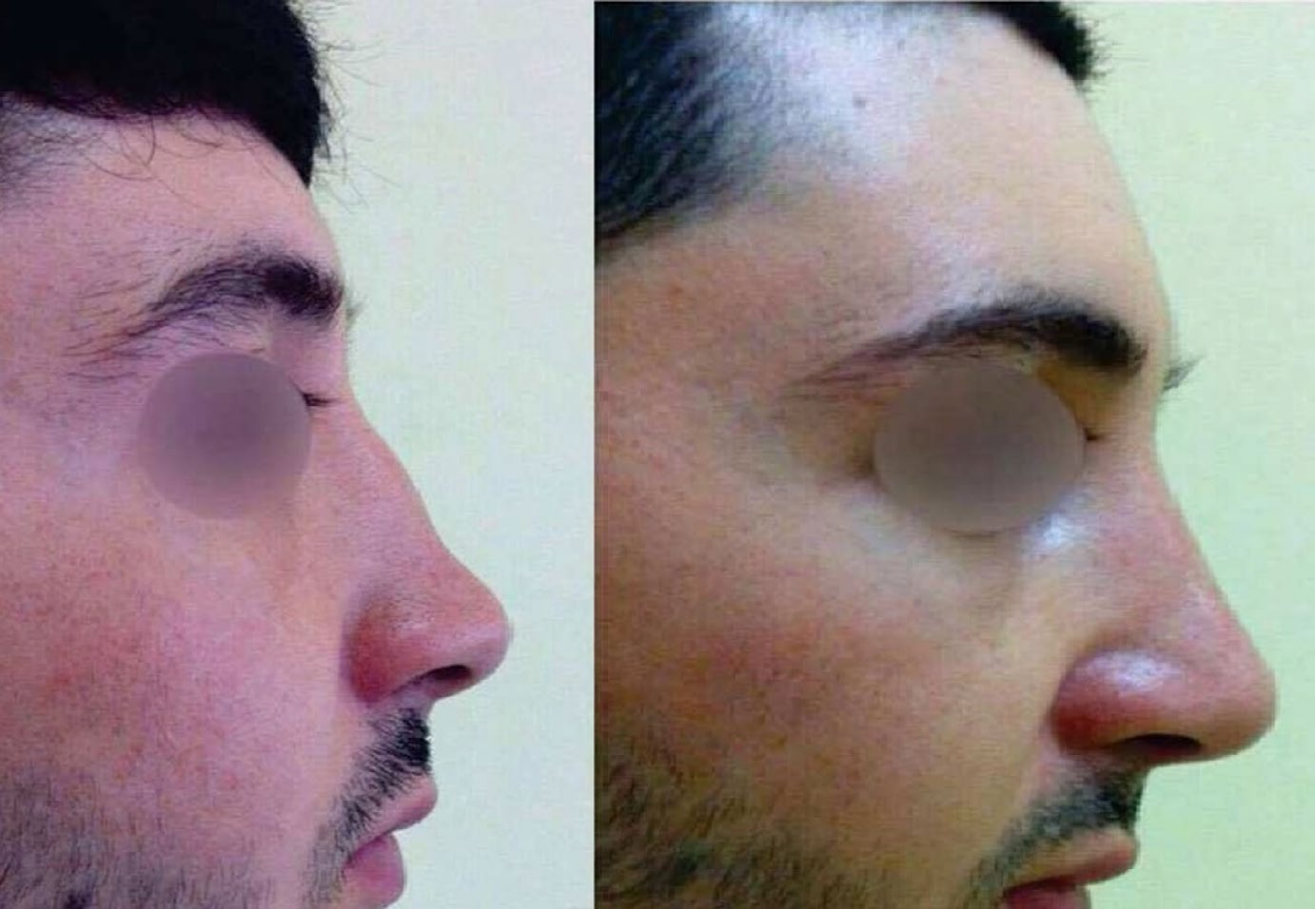
Preoperative and postoperative lateral views of a patient before dorsal nasal rhinoplasty using nasal septum allogenous cartilage graft.

**FIGURE 4:**
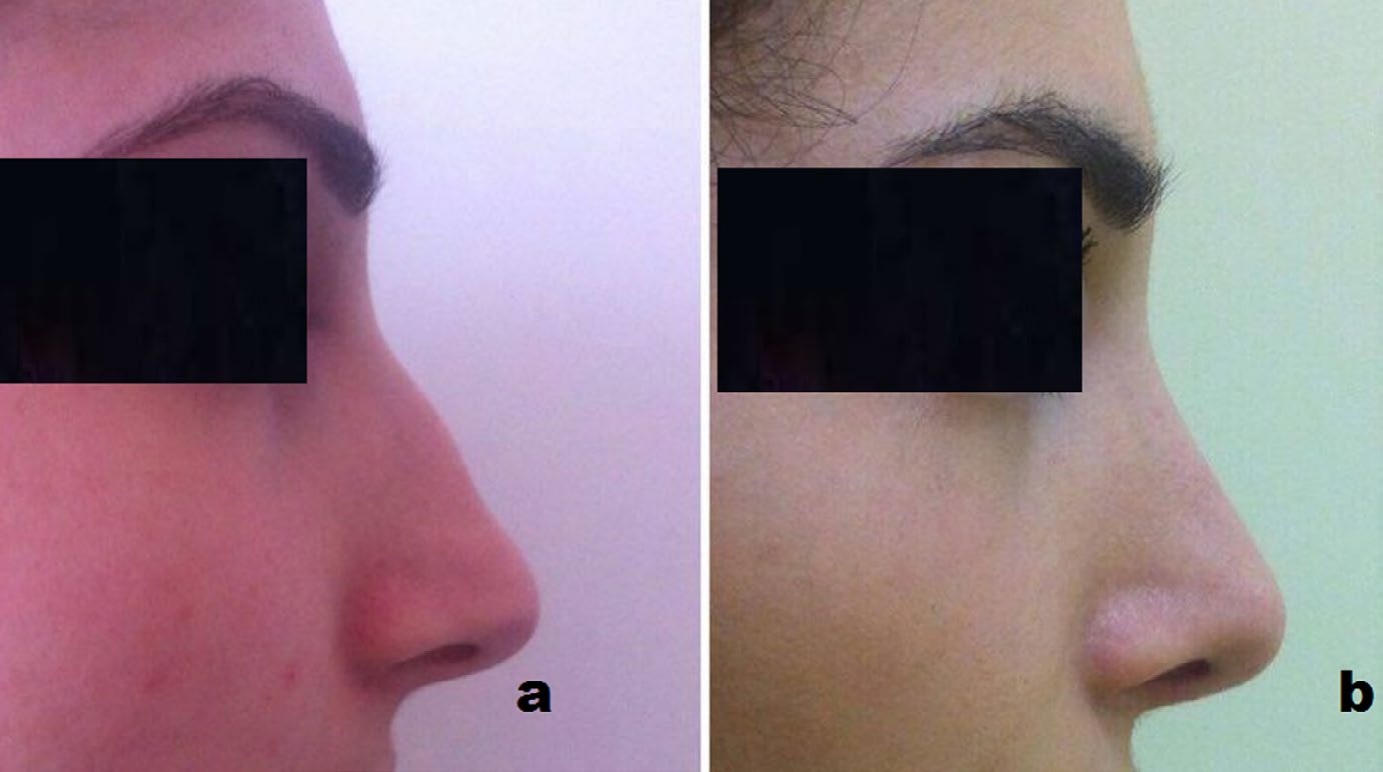
Preoperative and postoperative lateral views of a patient after dorsal nasal rhinoplasty using nasal septum allogenous cartilage graft.

**FIGURE 5:**
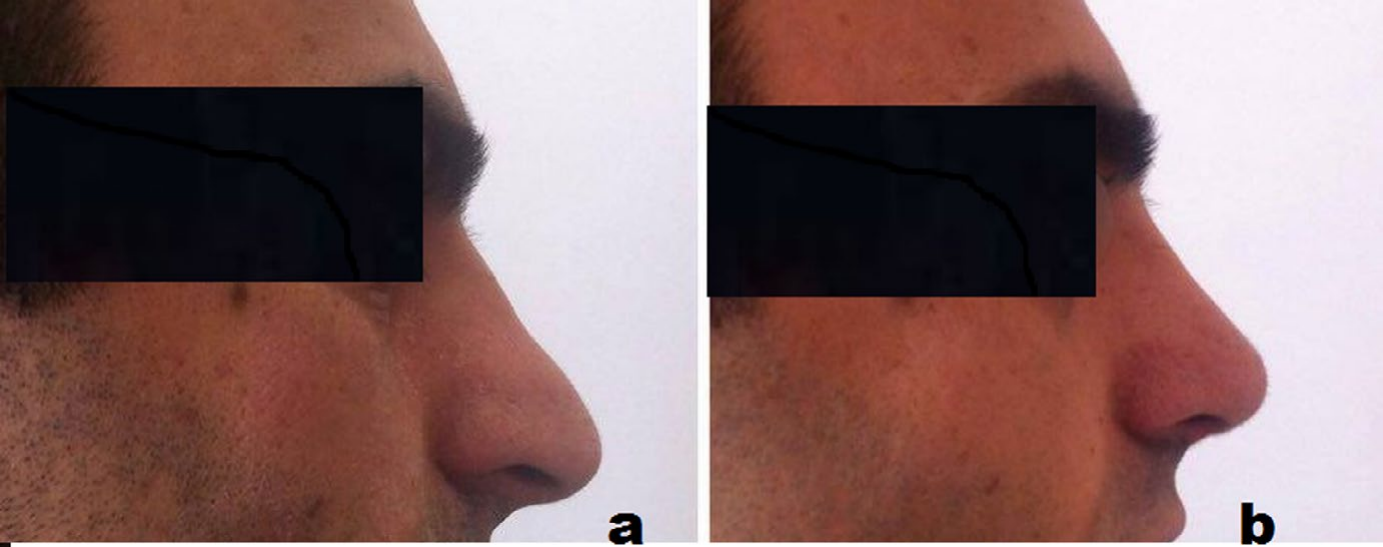
Preoperative and postoperative lateral views of a patient after dorsal nasal rhinoplasty using nasal septum allogenous cartilage graft.

At 1 year postoperatively, patients have adequate nasal forma using septal cartilage allografts (Figures 3, 4, 5).

In the 51 patients of Group II, one patient had transient tip paresthesia, one had minimal wing depression, one had a minimal resorption, and two patients had a leftward deviation of their dorsum at the 3‐month follow‐up and underwent revision.

**FIGURE 6:**
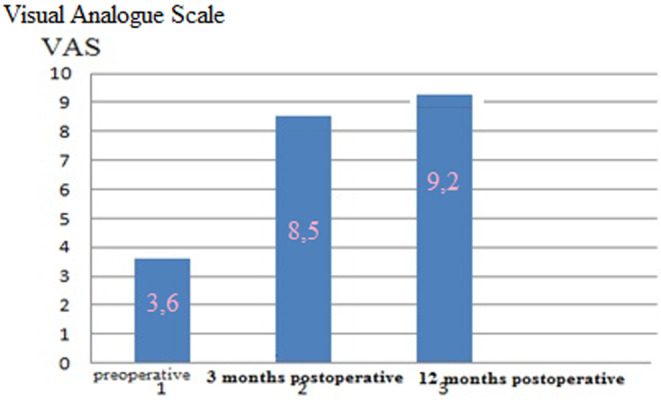
Preoperative and postoperative mean esthetic scores of Group I patients according to visual analog scale (VAS).

After rhinoplasty in the Group I patients, the mean score of VAS esthetic satisfaction with nose improved from 3.6 preoperatively to 8.5 3 months postoperatively and 9.2 12 months postoperatively (Figure 6).

**FIGURE 7:**
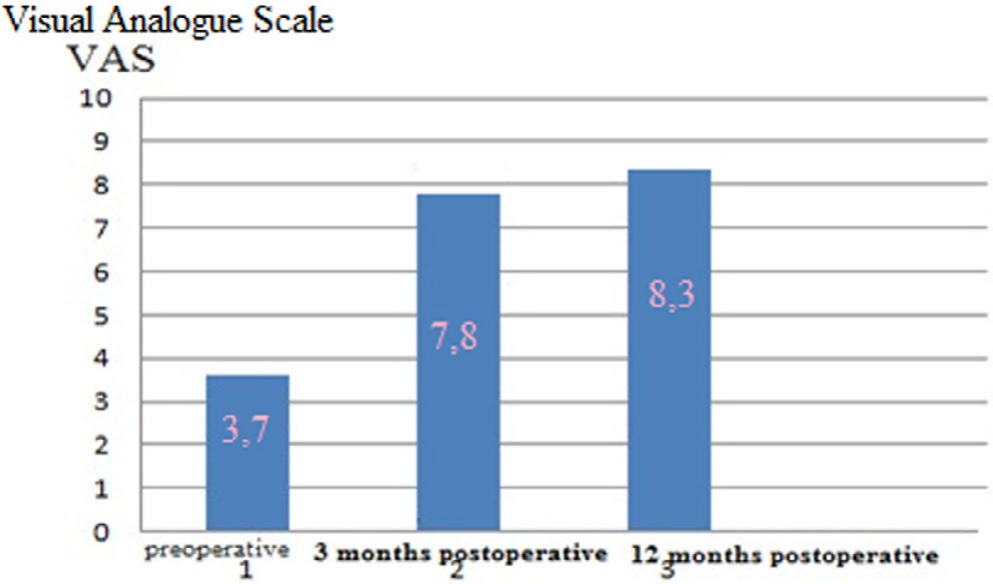
Preoperative and postoperative mean esthetic scores of Group II patients according to visual analog scale (VAS).

After rhinoplasty in the Group II patients, the mean score of VAS esthetic satisfaction with nose improved from 3.7 preoperatively to 7.8 3 months postoperatively and 8.3 12 months postoperatively (Figure 7).

## 4 Disscusion

Surgical rhinoplasty methods are constantly being improved, and new rhinoplasty methods are being offered [45]. The need for an optimal graft for cosmetic rhinoplasty procedures motivates the search for a biocompatible graft equivalent to nasal cartilage [46]. Many reviews discuss various graft materials used for dorsal rhinoplasty, discussing the advantages and disadvantages of each material, as well as the technical nuances of their use in clinical practice.Usually cartilage is taken from the septum, auricle, or rib. Each cartilage type has different specific characteristics and may be better suited to a particular clinical situation. Of the various graft options, septal cartilage has many positives and is the preferred choice for rhinoplasty [47]. Septal cartilage has a low percentage of resorption, extrusion, and deformation.

Auricle cartilage is also used in rhinoplasty to correct the collapse of the internal and external nasal valves [48, 49]. The disadvantage of auricle cartilage is that due to its natural curvature, it is difficult to form the desired contour to achieve the profile of the back of the nose; in addition, due to its internal memory and tendency to resorption, surface and contour irregularities develop over time in the case of ear cartilage wear.

An important aspect is the optimal configuration of the graft during its processing and modeling, which maximizes stress distribution and is able to provide support function in the distant period. Its displacement can be regarded as a disadvantage surgical technique, which in the future can lead to discrediting this type of cartilage graft [50].

To restore the esthetic shape of the nose, costal cartilage is traditionally used, which ensures an increase in the dorsum of the nose; however, the pain of the donor site (i.e., potential complications) forces many specialists to refrain from using this material [12, 51].

Recently, minimally invasive procedures to change the appearance of the nose have become widely accepted, techniques that have become to be known as NSR [40]. Non‐surgical approaches involve changing the appearance of the nose using filler injections, which are intended to replace surgical techniques [52, 53]. Scientific publications report the use of injectable cartilage in cosmetic rhinoplasty [18]. The long‐term clinical outcome of the study by Manafi et al. [17] supports the conclusion that the cartilage microstructure is not degraded when prepared as an injectable cartilage shave, but the authors note that more comprehensive long‐term In Vivo survival studies are needed to prove its efficacy.

There is an innovative method that involves mechanical centrifugation of cartilage [54].

Scientific developments in recent years among innovative methods offer regenerative strategies.

In addition to surgical techniques, tissue engineering strategies are being explored with using autologous chondrocyte micrografts obtained according to the Rigener protocol.According to the research by Ceccarelli et al. [55] the use of an autologous micrograft gives promising results and allows safe use for defects of nasal cartilage tissue.

Currently, lipofilling is used to increase volume, camouflage irregularities, and/or improve the skin and soft tissue. The fat graft has a regenerative potential that helps improve the quality of soft tissue.

One method of NSR is the use of fat grafting as a filler. However, there are a few cases in which patients have had serious complications, such as permanent blindness after fat embolism and respiratory diseases [56].

To optimize the postoperative period, PRF is now widely used because its clinical effects on tissue healing have been determined in many clinical procedures. It is associated with soft tissue regeneration by facilitating angiogenesis, including cell recruitment, proliferation, remodeling, and differentiation [57].

Although there are many reports in the literature about the effective use of PRF in dermatocosmetology, there is a limited number of studies on the use of PRF alone in dorsal rhinoplasty without cartilage or mixed with high‐density fat, and therefore, it is difficult to assess its effectiveness unequivocally [58].

PRF is an autologous concentrated blood derivative containing growth factors that accelerate tissue healing. Growth factors in PRF affect cell proliferation, differentiation, and migration; TGF‐1 enhances proliferation; PDGF promotes mesenchymal cell migration and survival; IGF inhibits cell death, VEGF initiates and stimulates angiogenesis; and EGF promotes cell proliferation and differentiation. Studies have shown the effectiveness of PRF in reducing the absorption of crushed cartilage in the nasal dorsum and increasing its survival. PRF is primarily used in diced cartilage to create camouflage or a slight dorsal augmentation and promotes tissue healing on the path to regenerative rhinoplasty. PRF reduces the rate of resorption of diced cartilage on the nasal dorsum by increasing its viability or shape retention. PRF has a positive effect on reducing inflammation in the postoperative period, promoting the healing process and can increase the viability of cartilage.

The main risks for nasal grafts are infection, extrusion, distortion, and resorption.

Among the complication dorsal rhinoplasty are irregularities along the nasal dorsum. In rhinoplasty, to avoid complications, it is necessary to maintain or reconstruct the stability, size, and position of the caudal cartilage.

Unlike other tissues, cartilage lacks vascularization and innervation; histologically, cartilage consists of ground substance and collagen and elastin fibers, and chondrocytes are located in the lacunae of the extracellular matrix. It is precisely this avascularity that determines the “immune privilege” of cartilage, which limits the immune system in recognizing and rejecting cartilage allografts.

Resorption of all types of cartilage remains a problem. In rhinoplasty, resorption of 20%–30% of the graft volume of autologous cartilage grafts of the nasal dorsum is a problem. If the transplanted cartilage is intact, it is protected from immunological recognition and destruction of the graft, which may be the most important factor associated with resorption. Excessive trauma to the cartilage cell matrix may be detrimental to its viability. Significant resorption can be expected when using diced cartilage due to the large surface area compared to whole cartilage. To preserve the volume of the cartilage graft, it is not recommended to crush it so as not to disrupt the permanent integrity and proliferation of chondrocytes.

The allograft located on the bone portion is nourished by the receptor bone; osteoclasts migrate through the Haversian canals in the allograft, resorbing the allograft and releasing the embedded bone morphogenetic protein and growth factors, which then transform into osteoblasts and ultimately into osteocytes [59].

The irradiation process has a negative effect on the viability of the graft; however, there are reports in the literature showing that irradiated cartilage has comparable complications rates with autologous grafts [60].

Currently, irradiated cartilage allografts are widely used in cosmetic rhinoplasty. Cartilage allografts are optimal grafts for rhinoplasty, with good cosmetic effect and low complication rate; there is only one surgical site and low antigenicity due to the isolation of chondrocytes in lacunae [29]. In the biochemical content of the septal nasal cartilage, by dry weight, 90%–95% includes collagen II. It also contains a small amount of collagen I, IX, X, and XI.

One of the specific complications when using cartilage allografts is their resorption. This process occurs due to the death of the main cells of the cartilage tissue—chondrocytes, which ensure the structural integrity of the elastic and hyaline cartilage [61]. It has been reported that the rate of resorption of irradiated homologous costal cartilage (31%) is significantly higher than that of autologous costal cartilage (3%), which may be due to the absence of viable cells in the treated graft tissue and its subsequent inability to remodel In Vivo [62].

AlloDerm is a lyophilized sheet of collagen matrix from human dermis and allows for host tissue ingrowth. AlloDerm for the dorsal magnification with this material is ∼3 mm^2^; in some reports, implantation rates decrease to less than 50% at 3 months after surgery [30].

For large defects of the dorsal part, fresh frozen allograft cartilage is also used; however, partial absorption is definitely a disadvantage [63, 64, 65].

Various alloplastic materials are also used in esthetic rhinoplasty; however, the disadvantage of alloplastic materials is the formation of a fibrous capsule, which can lead to capsular contracture [66].

The disadvantage of alloplastic materials is fibrous capsular formation, which can lead to capsular contracture [67].

Currently, rhinoplasty using HA has become a popular procedure due to its effectiveness and short recovery time; however, the indications, technique, long‐term results, and results are still being discussed [40, 68]. HA filler injections are not without complications, and blindness and skin necrosis have been reported as a result of nasal filler injections, injection site reactions, infections, hypersensitivity, vascular disorders, and so forth [69, 70, 71]. The current situation makes the issues of developing a concept for the use of an allogeneic nasal cartilage transplant, depending on the clinical case, and also the optimal method of its use for reconstruction of dorsal defects of the nose.

This article reports the long‐term efficacy use of the allogeneic nasal cartilage in the reconstruction of the nasal dorsum. In this study, objective and subjective assessments of the results were carried out up to 5 years after surgery; success was achieved in 104 cases.

As patient satisfaction is the primary measure of success in facial cosmetic surgery, the VAS was included in this study. Results from Likert questions indicated patient satisfaction after dorsum augmentation.

Evaluation of the results of the operations performed with the use of an allogeneic nasal septal cartilage graft showed the low antigenicity of the cartilaginous tissue. The use of this material made it possible to achieve stable positive esthetic and functional results.

Allogeneic nasal cartilage graft undergoes minimal changes and its viability is maintained by storage at a temperature of 4°C. Under optimal conditions of sterilization, conservation, and storage, it does not undergo resorption, retains its strength characteristics, and provides a stable result in the long‐term postoperative period.

Considering safety and feasibility of allogeneic nasal cartilage in our study, it was established that it is a valuable alternative to autologous cartilage for patients undergoing dorsal rhinoplasty, because of fewer incisions and scars on our patients. The use of allogeneic cartilage of the nasal septum in our study gave promising esthetic results without any side effects. The use of PRF in combination with allogeneic nasal septal cartilage had a positive effect on wound healing, reduced postoperative edema, and improved esthetic results. The esthetics were significantly more pronounced in Group I than those in Group II (control group).

Selection criteria for allogenic nasal cartilage graft for esthetic rhinoplasty of the dorsal nasal part, such as microstructure, biochemical composition, and mechanics, contribute to good results of the procedure with the least resorption of the graft.

Limitations of the study include the sample size and short follow‐up period. Further study comparing clinical and histological results will advance our understanding of the long‐term effectiveness of these cartilage grafts.

## 5 Conclusions

Rhinoplasty with allogeneic cartilage graft of the nasal septum allows to achieve stable, positive, functional, and esthetic results and is safe to use. It is an alternative to autologous cartilage in the rhinoplasty and prevents complications and additional surgical procedures.

## Author Contributions

A.H. designed the work; acquired, and analyzed data; drafted, revised, and approved the manuscript; and agreed to be accountable for all aspects of the work.

## Ethics Statement

The study was reviewed and approved by the local ethics committee and was in accordance with the norms of the World Medical Association and the Helsinki Declaration.

## Consent

The informed consent was obtained from all the individual participants included in the study and accompanying images. Consent for publication: Patients were informed verbally and in writing about the study, and gave written informed consent.

## Conflicts of Interest

The authors declare no conflicts of interest.

## Data Availability

All data generated or analyzed during this study are included in this published article.
